# Rations for Hospitals

**Published:** 1918-05-04

**Authors:** 


### RATIONS FOR HOSPITALS.

### An Authoritative Diet Scale.

It was early recognised, when compulsory rationing was introduced, that some special provision must be made for institutions, more especially voluntary hospitals. The restoration to health of the patients was a matter of such importance that the provision of their food could not be left to chance, and the Food Controller at once took the matter in hand, and very wisely consulted hospital authorities.

With the assistance of an Advisory Committee of hospital managers on which sat three or four experienced hospital managers, two of the leading physiologists, and with the aid of practical housekeepers, a scale was drawn up fixing requirements of the average per head necessary in a civil general hospital.

In drawing up this scale, the Advisory Committee was bound to adhere to the strict maximum fixed by the Food Controller in regard to controlled articles, such as meat, sugar, and fats; but at the same time it was fully recognised that sick people wanted special nourishment, such as they could easily assimilate, and that those who looked after them and were constantly exposed to infection, and whose places, if incapacitated, could not be filled in an emergency, must be specially nourished.

The result of the deliberations has been the drawing up of the scale, a summary of which follows.

In view of the difficulty which may exist from time to time in securing fish, poultry, or game, this scale has been amended both for hospital patients and for the resident staff by a permission, as long as supplies are available, to substitute when considered necessary a further 8 oz. of bacon or ham for the 32 oz. of fish, poultry, or game.

In interpreting and working on this scale, hospital managers should realise that an attempt has been made to formulate an average supply on which they may requisition for the full number of patients in the wards, some of whom may require extra supplies of some special articles of diet, others may not require so much. But it has been found in practice that for an average-sized general hospital with an average staff this scale is satisfactory. It behoves all, therefore, to attempt to work on this scale loyally. The rationed articles they cannot get more of, with regard to the other articles of diet enumerated, they will be playing the game by adhering generally to the scale, and this we fully believe they will do in the interests of their own institution, in the interest of those for whom they cater, and in the interest of the outside public.

It may be added that in their form R.M. 40, publishing this special institutional ration scale for hospitals and sanatoria, special scales are also published for naval and military hospital patients, and for tuberculosis cases, and we understand that a scale is in preparation also for special children's hospitals. The children who may be in general hospitals are, of course, included in the average patients.

THE INSTITUTIONAL DIET SCALE.

This scale is applicable to the residents and professional staffs of hospitals, including the nursing staff. The allowances for one person per week in each case are as follows:

Meat, 1 lb.; bacon (boneless), 4 oz.; milk, 8.4 pints; bread, 70 oz.; potatoes, 70 oz.; sugar, 8 oz.; butter or margarine, 4 oz.; cheese, 4 oz. (should be used as little as possible); rice, 8 oz.; jam, 8 oz.; oatmeal and pulses, 4 oz.; fish (fresh) or poultry or game, 2 lb. (for this 8 oz. of bacon may be substituted if available); sardines or other dried or preserved fish, 4 oz.; vegetables (fresh), 1¾ lb.; eggs, 4.

This diet scale yields an average per day per person of 3.45 oz. of protein and 2,462 calories.

